# Connexin 50 Expression in Ependymal Stem Progenitor Cells after Spinal Cord Injury Activation

**DOI:** 10.3390/ijms161125981

**Published:** 2015-11-06

**Authors:** Francisco Javier Rodriguez-Jimenez, Ana Alastrue-Agudo, Miodrag Stojkovic, Slaven Erceg, Victoria Moreno-Manzano

**Affiliations:** 1 Neuronal and Tissue Regeneration Laboratory, Centro de Investigación Príncipe Felipe, Valencia E-46012, Spain; aalastrue@cipf.es (A.A.-A.); vmorenom@cipf.es (V.M.-M.); 2 Spebo Medical, 16000 Leskovac, Serbia; mstojkovic@spebo.co.rs; 3 Human Genetics, Faculty of Medical Sciences, University of Kragujevac, 34000 Kragujevac, Serbia; 4 Stem Cell Therapies in Neurodegenerative Diseases Laboratory, Centro de Investigación Príncipe Felipe, Valencia E-46012, Spain; serceg@cipf.es

**Keywords:** connexins, spinal cord, ependymal stem progenitor cells

## Abstract

Ion channels included in the family of Connexins (Cx) help to control cell proliferation and differentiation of neuronal progenitors. Here we explored the role of Connexin 50 (Cx50) in cell fate modulation of adult spinal cord derived neural precursors located in the ependymal canal (epSPC). epSPC from non-injured animals showed high expression levels of Cx50 compared to epSPC from animals with spinal cord injury (SCI) (epSPCi). When epSPC or epSPCi were induced to spontaneously differentiate *in vitro* we found that Cx50 favors glial cell fate, since higher expression levels, endogenous or by over-expression of Cx50, augmented the expression of the astrocyte marker GFAP and impaired the neuronal marker Tuj1. Cx50 was found in both the cytoplasm and nucleus of glial cells, astrocytes and oligodendrocyte-derived cells. Similar expression patterns were found in primary cultures of mature astrocytes. In addition, opposite expression profile for nuclear Cx50 was observed when epSPC and activated epSPCi were conducted to differentiate into mature oligodendrocytes, suggesting a different role for this ion channel in spinal cord beyond cell-to-cell communication. *In vivo* detection of Cx50 by immunohistochemistry showed a defined location in gray matter in non-injured tissues and at the epicenter of the injury after SCI. epSPCi transplantation, which accelerates locomotion regeneration by a neuroprotective effect after acute SCI is associated with a lower signal of Cx50 within the injured area, suggesting a minor or detrimental contribution of this ion channel in spinal cord regeneration by activated epSPCi.

## 1. Introduction

Connexins are a family of trans-membrane proteins whose major function is gap junction intercellular communication (GJIC), which allows transfer of important metabolites, ions or small molecules (<1 kDa). Six connexins form a connexon or hemichannel, and two connexons from adjacent cells make a full intercellular gap junction channel that allows direct communication between cells. In addition, undocked hemichannels at the cell surface allow cells to exchange ions and other molecules across the cell membrane with the extracellular space. Concerning the expression of Cxs in differentiated cells from the central nervous system (CNS), there are many gap-junctions between astrocytes, fewer between oligodendrocytes and astrocytes, and few or none between oligodendrocytes or between neurons and glia [1]. In the spinal cord, astrocytes in gray matter are generally strongly coupled by Cxs, whereas astrocytes in white matter show diverse coupling [2]. Oligodendrocytes express Cx29, Cx32, Cx45, and Cx47 [1] and are differentially expressed in spinal cord tissue. For instance, Cx32 and Cx47 are mainly found in white matter tracts, while Cx29 is more robust in gray matter [1]. There are differences in coupling efficiency between spinal cord oligodendrocytes from gray and white matter [3]. Astrocytes express Cx26 [4], Cx30 [5] and Cx43 [6,7]. Cx30 is localized primarily to gray matter [5,8] and can restrict survival of newborn neurons [9]. Cx43 is localized in white and gray matter [10]. Cx43 forms part of neuron-glia interactions and impairs neuronal plasticity [11]. The most frequent sub-cellular location for Cxs is at the plasma membrane. However, Cxs have also been detected within the cytoplasmic region like (Cx26 and Cx43) [12,13], or in the nucleus (Cx43 and Cx30) [13,14]. The nuclear location has been suggested to be associated with regulation of gene expression that may control cell differentiation by mechanisms associated or not to GJIC [12,14,15]. The major strategies for SCI regeneration are activation of endogenous neural stem cells, and cell transplantation therapies. In both strategies, it is crucial to understand the underlying mechanisms of maintenance, activation, and differentiation of neural stem cells and the subsequent processes, including the migration, survival, and functional maturation of differentiated cells. In the last few years the role of Cxs in crucial processes of stem cells, such as self-renewal and differentiation has increased the interest of researchers involved in the stem cell field. Some studies have suggested a possible role of Cxs in cell-cell communication during differentiation of neural progenitors [16,17]. Although, GJIC is required to maintain self renewal of stem cells [18], Cxs might contribute to the differentiation process of stem cells beyond cell communication [19]. Regardless, the role of Cxs in the differentiation process of neural stem cells toward cells of neuronal lineage is poorly understood. In neural stem cells grown as neurospheres, over-expression of Cx36 increased the number of neurons and oligodendrocytes derived from the modified neural stem cells [20]. Cxs also have a relevant role in neurogenesis and in the myelin function in the CNS. The relevance of Cx43 in spinal cord neurogenesis has also been reported [21] as well as in remyelinating/recovering spinal cord tissue [22]. In fact, mutations in *Cx* genes have detrimental effects on myelin function of astrocytes and oligodendrocytes [1]. Cxs play an important role in normal spinal cord physiology [23,24,25,26], as well as after injury [27]. Despite extensive literature concerning the role of Cxs in the neuronal lineage, the regulation and differentiation of neural stem cells [16,20,28] and their contribution in spinal cord regeneration, our knowledge of Cx50 expression in these processes is limited.

Transplantation of GFP-epSPCi immediately after SCI favors functional locomotor regeneration [29,30] and is associated with reduced expression of Cx50 at the injured and engrafted area. In fact, epSPCi, activated by the injury, showed decreased Cx50 expression levels. Cx50 appears to be a marker for astrocytes and oligodendrocytes in epSPC-derived cells showing a preferential role for glial differentiation while inhibiting neuronal differentiation. Taken together, our results suggest a minor or detrimental contribution of Cx50 in spinal cord injury regeneration. 

## 2. Results

### 2.1. Expression of Cx50 in epSPC and epSPCi under Spontaneous Differentiation Conditions

**Figure 1:**
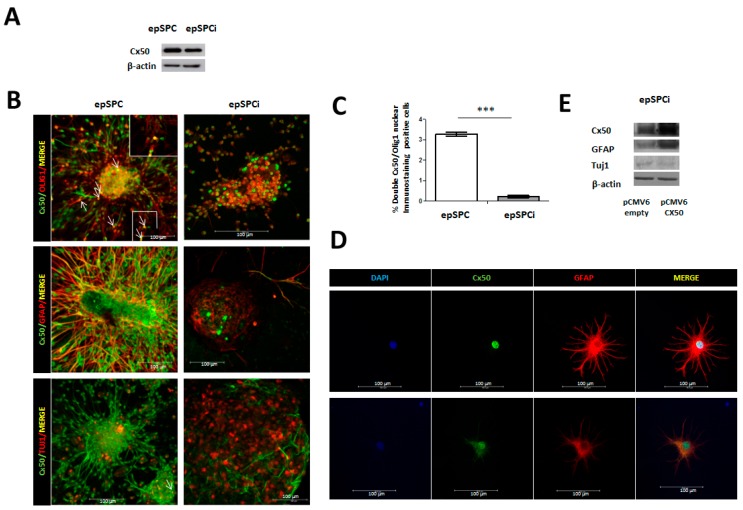
Cx50 expression in epSPC compared to epSPCi and their derived differentiated cells. (**A**) Cx50 exhibited higher protein expression in epSPC compared to activated epSPCi; (**B**) Immunocytochemical analysis of Cx50 simultaneously with the markers for the neuronal lineage (GFAP for astrocytes, Olig1 for oligodendrocytes and Tuj1 for neurons) in epSPC and epSPCi under spontaneous differentiation conditions for 24 h. The inset shows a *zoom in* area for easier visualization of Olig1 and Cx50 co-localization. Arrows point double-stainings. Scale bar = 100 µm; (**C**) Quantification of co-localization for Cx50 and Olig1; *** *p* < 0.001 *vs.* epSPC; (**D**) Immunocytochemistry of Cx50 and GFAP antibodies in primary culture of mature astrocytes isolated from non-injured spinal cord rats. Scale bar = 100 µm; (**E**) Modulation of GFAP and Tuj1 in epSPCi after over-expression of Cx50 by using pCMV6-Cx50 expression vector in comparison to pCMV6-empty (control).

We determined by Western blotting and Immunocytochemical analysis that epSPC showed higher Cx50 protein expression compared to epSPCi (epSPC activated by injury) (Figure 1A,B). epSPC and epSPCi were grown under suitable conditions to force their spontaneous differentiation to cells of neuronal lineage (oligodendrocytes, astrocytes, and neurons), as previously described (Materials and Methods). Immunocytochemical analysis of Cx50 showed co-localization with the oligodendroglial lineage-associated marker Olig1 at the nucleus of both epSPC and epSPCi. epSPC exhibited a significantly higher number of cells that co-expressed Cx50 (green signal) and Olig1 (red signal) at the nucleus in comparison with activated epSPCi (3.28 ± 0.101 *vs.* 0.23 ± 0.053, *n* = 3) (*p* ≥ 0.001) (Figure 1B). Cx50 (green signal) was highly colocalized with the astrocytic marker GFAP (red signal) at the cytoplasmic region (Figure 1B). Again, nuclear staining was detected for Cx50 in GFAP positive cells. Some GFAP positive epSPC or epSPCi cultures also presented Cx50 expression in both nucleus and plasma membrane and some of them only at the nucleus (Figure 1B). Decreased global expression of Cx50/GFAP positive cells was observed in activated epSPCi in comparison to epSPC (Figure 1B). Moreover, isolation of mature rat astrocytes from healthy spinal cord tissue confirmed that Cx50 is present at the nucleus (labeled by DAPI) of GFAP positive cells (Figure 1D; upper pictures). However, some astrocytes express Cx50, not only at the nucleus but also at the plasma membrane establishing differences within the astrocyte population (Figure 1D; lower pictures). Finally, we detected a lower signal for the neuronal marker Tuj1 but higher for Cx50 in epSPC in contrast with activated epSPCi. No co-localization of Cx50 and Tuj1 was observed in either epSPC or epSPCi (Figure 1B). Transfection of activated epSPCi with pCMV6-empty as control or pCMV6-Cx50 to force Cx50 expression was performed for 48 h and subsequently cultured under spontaneous differentiation conditions for 24 h. Thus, induced expression of Cx50 by pCMV6-Cx50 was found in parallel to increased GFAP expression. In contrast, the opposite expression was observed for the neuronal marker Tuj1, with lower levels of protein expression associated with increased Cx50 expression (Figure 1E).

### 2.2. Cx50 Expression in Directed-Differentiation Process of epSPCs to Mature Oligodendrocytes

**Figure 2:**
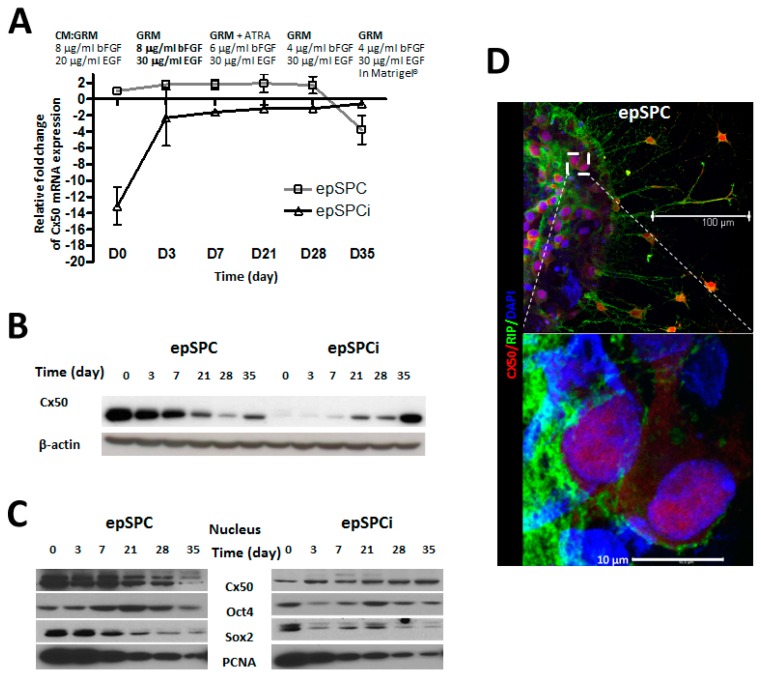
Evolution of Cx50 mRNA expression of epSPC and epSPCi during directed differentiation to oligodendrocytes. (**A**) Changes in Cx50 mRNA expression determined by qRT-PCR during directed differentiation to oligodendrocytes; (**B**) Changes in total Cx50 protein expression of epSPC and epSPCi during directed differentiation to oligodendrocytes; (**C**) Subcellular fractionation of proteins from epSPC and epSPCi shows evolution of nuclear expression of Cx50, Oct4 and Sox2 during directed differentiation to oligodendrocytes. PCNA was used as differentiation control; (**D**) Immunostaining of Cx50 and mature oligodendrocyte marker RIP in epSPCi after 21 days of differentiation. Scale bar = 100 µm (**upper** panel); 10 µm (**lower** panel).

Spontaneously differentiated epSPCs induced the generation of an oligodendroglial population which expressed both, nuclear Cx50 and the oligodendroglial lineage-associated marker Olig1 (Figure 1A). Therefore, we conducted a directed-differentiation process to oligodendrcyte to monitor the Cx50 location at different maturation stages by qRT-PCR and Western blotting. At the transcriptional level, the tendency of expression of Cx50 in epSPCi differs with epSPC from the beginning and along oligodendrocyte differentiation. Unlike the expression in epSPC, Cx50 mRNA progressively increased in activated epSPCi during directed differentiation to mature oligodendrocytes (Figure 2A). Opposite expression of total Cx50 protein was observed in epSPC and epSPCi during directed differentiation (Figure 2B). Sub-cellular Cx50 protein fractionation to separate nuclear fractions demonstrated an opposite profile, since nuclear Cx50 decreased during directed differentiation to mature oligodendrocytes in contrast to the increased expression in epSPCi. The protein expression of the pluripotency markers Oct4 and Sox2 in both epSPC and epSPCi is significantly reduced at the end of the directed-differentiation process (Figure 2C). PCNA was included as positive control, showing a diminishing expression in concordance with the acquisition of a mature non-proliferative state. Nuclear Cx50 localization was confirmed by immunofluorescence and confocal microscopy analysis at day 21 of the differentiation process. The marker for mature oligodendrocyte, RIP was used as positive control of mature oligodendrocyte differentiation, detected on the cell membrane of cells with a positive nuclear signal for Cx50 (Figure 2D).

### 2.3. Cx50 Expression after Spinal Cord Injury and epSPCi Transplantation

**Figure 3:**
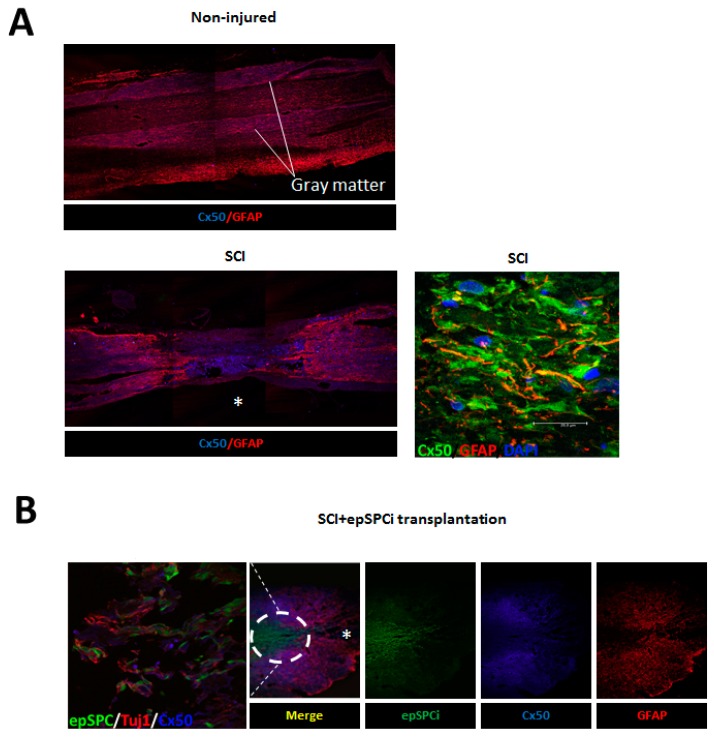
Cx50 expression in healthy spinal cord tissue, after SCI, and epSPCi transplantation. Immunohistochemical evaluation of Cx50 and the astrocytic marker GFAP in tissues from (**A**) non-injured spinal cord and 2 months after SCI (Zoom; Cx50/GFAP/DAPI); Scale bar = 25 µm, and (**B**) 2 months after SCI with GFP-epSPCi transplantation (Zoom; epSPCi/TuJ1/Cx50); * epicenter of the injury area.

Immunolocalization of Cx50 *in vivo* displayed ubiquitous distribution in the non-injured spinal cord with a significant presence in gray matter (Figure 3A). Two months after SCI, Cx50 expression was increased within the injured area delimited by the GFAP signal surrounding the glial scar (Figure 3A). Co-localization of GFAP and Cx50 was visualized at the injured area *in vivo* after SCI (Figure 3A, zoom). Activated GFP-epSPCi were transplanted immediately after SCI and animals were sacrificed two months later. Previously, we showed that epSPCi acute transplantation significantly improves locomotion recovery [29,31]. Immunohistochemical evaluation of the spinal cord tissue showed that the area invaded by the GFP-epSPCi transplanted cells exhibited profuse neuronal projections stained by Tuj1 (Figure 3B; Zoom), but poor or absent expression of GFAP and Cx50 (Figure 3B).

## 3. Discussion

In the adult mammalian spinal cord there is limited neurogenesis despite the presence of ependymal neural stem progenitor cells (epSPC) [32]. However, after spinal cord injury (SCI) the epSPC population is activated (epSPCi) thereby increasing its neurogenic potential [33,34].

After SCI, undifferentiated epSPCi in the vicinity of the lesion epicenter vigorously proliferate, migrate to the injured region, and differentiate into astroglia or oligodendrocyte precursor cells but show modest neuronal differentiation [33,34]. Furthermore, the epSPCi, either endogenous or ectopically transplanted into the injured spinal cord, provide trophic support, help restore neuronal connectivity, and facilitate neuronal regeneration [29,30].

Connexins are differentially expressed in specific tissues and cells of the CNS [1,35] and may exert redundant but also independent actions in stem cell renewal and differentiation. Here we show for the first time, that Cx50 is expressed in the neuronal precursor cell population derived from the adult spinal cord (epSPC). In addition, we found that the expression level of Cx50 is significantly reduced in epSPCi, the neural precursor cells activated by SCI in comparison with epSPC in non-injured tissue. This protein expression profile could confer a different behavior and regeneration potential within both cell populations, for instance in conditioning their neuronal lineage destination. Under spontaneous differentiation conditions, Cx50 and GFAP were highly expressed at the cytoplasmic membrane of epSPC-derived astrocytes, but in epSPC-derived oligodendrocytes they were only expressed in the nuclear region. In contrast, the Cx50 immune signal was nearly undetectable in cells derived from activated epSPCi and showed significantly less co-localization with the glial lineage-associated markers GFAP and Olig1. Co-localization of Cx50 and Tuj1 was not observed in either epSPC or epSPCi dismissing Cx50 for coupling neurons with other cells of neuronal lineage. Connexins are mainly located at the plasma membrane, however, we have shown their presence in the nucleus thus confirming their localization is not strictly limited to this location. In accordance, under certain pathological conditions such as cancer, Cx26 and Cx43 have been detected in the cytoplasm [12,13] and Cx43 and Cx30 in the nucleus [13,14]. Nuclear localization of connexins may be associated with the regulation of gene expression controlling growth [36] and differentiation by mechanisms in addition to, but different from GJIC [12,14,15]. Moreover, immunodetection of Cx50 in isolated mature astrocytes from spinal cord also showed two different sub-cellular sites that suggests different actions for this ion channel depending on its location in the cells. Over-expression of Cx50 in epSPCi under differentiation conditions resulted in increased GFAP expression but decreased Tuj1 suggesting that Cx50 may favor astroglial and impair neuronal differentiation. In fact, high expression of Cx50 in epSPC and low expression of Tuj1 contrasts with that in activated epSPCi (Figure 1). Astrogliosis contributes to form the glial scars after SCI and disrupts regeneration of neuronal axons [33]. Promoting the generation of oligodendrocytes, which are myelin-forming cells, and disrupting astrocytes by transplanted neural stem cells, may facilitate sensory and locomotor recovery [37]. The decreasing expression of Cx50 in epSPC and the increasing expression of Cx50 in epSPCi during directed differentiation to oligodendrocytes, suggests additional functions for Cx50 beyond cell-to-cell communication. The increased expression of Cx50 in epSPCi during the oligodendrogenesis may indicate that Cx50 plays an integral role in the activation of epSPCi. However, the role of increased expression of nuclear Cx50 and whether it contributes to the increased plasticity and regeneration potential of epSPCi compared to epSPC remains to be elucidated. The precise expression profile of the pluripotency markers Sox2 and Oct4 in epSPC and epSPCi during directed differentiation suggests an orchestrated expression of key proteins plays an important role. The specific distribution of connexins in mature oligodendrocytes, astrocytes or neurons and their contribution in SCI pathology have already been explored for other members of this family of ion channels [1,35]. For instance, alterations in astrocytic Cx43 occurred in gray and white matter after SCI in rodents [38]. The authors concluded that spinal cord astrocytes become reactive by altered Cx43 expression as a direct result of traumatic SCI. Another study showed that astrocytic Cx43 was considerably increased four weeks after injury, while there were no changes in Cx32 or Cx36 [39]. However, little is known about the expression of Cx50 in stem cells and its contribution to their crucial biological processes in spinal cord regeneration. In fact, most knowledge regarding Cx50 has been focused in developmental anomalies associated with the lens as well as its relevance in cataract formation and other human eye disorders [40]. Cx50 is mainly expressed in gray matter wherein astrocytes are strongly coupled. According to the observed high assembly between Cx50 and GFAP it is plausible that Cx50 contributes actively to astrocyte connections in gray matter [2]. epSPCi transplantation in acute SCI rescues locomotor function and interestingly, transplanted epSPCi at the injured area do not express Cx50. It is known that transplantation of GFP-epSPCi improves locomotor recovery after SCI [29,31]. We observed that the region of hosted cells lacks Cx50 and GFAP expression in the grafted region, creating a more permissive environment for axonal survival and growth that favors the neural process by the epSPCi. These results suggest a minor or detrimental contribution of Cx50 in SCI regeneration by activated cells. The hypothesis of a putative harmful contribution has also been reported for other connexins, Cx43 plays a role in the spread of injury and its blockage leads to improved recovery [41]. Further studies aimed at elucidating the role of Cx50 in the differentiation of progenitor cells towards cells of the neuronal lineage will help to potentiate the endogenous regeneration machinery that coupled with advances in cell transplantation, may bolster the treatment of SCI and functional rescue. 

## 4. Experimental Section

### 4.1. Spinal Cord Contusion and GFP-epSPCi Transplantation

Female Sprague Dawley rats (~200 g) were divided into three groups: (a) Control: un-injured animals (*n* = 4); (b) SCI by severe contusion (*n* = 14); (c) epSPCi: SCI by severe contusion by applying 250 Kdyn at T8, with the Infinitive Horizon Impactor (IHI; Precision Systems, Louisvile, KY, USA), as previously described [29], followed by epSPCi transplantation (*n* = 13). The maintenance and use of all animals was in accordance with the National Guide for the Care and Use of Experimental Animals Committee (Animal Care Committee of the Research Institute Principe Felipe (Valencia, Spain) (Real Decreto 1201/2005). A total of 10^6^ GFP-epSPCi were transplanted by stereotaxis at a distance of 2-mm rostral and caudal to the lesion in four positions, from anterior to posterior. Post-surgery care of injured rats was performed as previously described [29]. All animals were sacrificed two months after injury and intracardially perfused with PFA 4% for spinal cord tissue dissection.

### 4.2. epSPC/epSPCi, GFP-epSPCi Isolation

epSPC, epSPCi or GFP-epSPCi were harvested from adult female Sprague Dawley rats, SD-Tg(GFP)2BalRrrc (Rrrc, University of Missouri Columbia, Columbia, MO, USA) [42], eGFP+/+ homozygote transgenic rats were used for cell transplantation experiments and eGFP−/− rats for *in vitro* assays. epSPC were obtained from a non-injured rat and epSPCi were isolated one week after severe contusion of spinal cord (250 kdyn at T8–T9). Cells were isolated and cultured as previously described in growth medium (DMEM/F12 supplemented with 100 units/mL penicillin, 100 µg/mL streptomycin, l-glutamine 2 mM, 5 mM Hepes buffer, 0.125% NaHCO_3_, 0.6% glucose, 0.025 mg/mL insulin, 80 µg/mL apotransferrin, 16 nM progesterone, 60 µM putrescine, 24 nM sodium selenite, 4 µg/mL BSA, heparin 0.7 U/mL, 20 ng/mL epidermal growth factor (EGF) and 20 ng/mL basic fibroblast growth factor 2 (bFGF) [29].

### 4.3. Spontaneous Differentiation of epSPC

For spontaneous differentiation process, Heparin and the mitogenic factors EGF and FGF were removed from the growth medium. BSA was replaced by 2% FBS. The culture plates were coated with Matrigel (BD Bioscience, San Jose, CA, USA). Forced spontaneous differentiation of epSPC and epSPCi was maintained for 24 h and afterwards the cells were harvested. Cells were fixed with 2% PFA and processed for immunocytochemical evaluation.

### 4.4. Cx50 over-Expression in epSPCi

Cx50 expression was analyzed in epSPCi after cell transfection with pCMV6 empty expression vector (control) (Ref. PS100001, Origene, Rockville, MD, USA) or with pCMV6-Cx50 expression vector (over-expression) (Ref. RR203586, Origene, USA) using Lipofectamine 2000 according to manufacturer’s instructions (Invitrogen, Madrid, Spain). Lipofectamine 2000 was removed 24 h after transfection. Then, cells were subjected to spontaneous differentiation for 24 h and harvested.

### 4.5. Oligodendrocyte-Directed Differentiation

Cell differentiation into oligodendrocyte precursor cells (OPCs) was performed as previously described [29]. Briefly, epSPC or epSPCi were cultured with glial restriction media (GRM): DMEM:F12, B27 supplement (Invitrogen), 25 µg/mL insulin, 6.3 ng/mL progesterone, 10 µg/mL putrescine, 50 ng/mL sodium selenite, 50 µg/mL holotransferin, 40 ng/mL tri-iodo-thyroidin, supplemented with 4 ng/mL bFGF and 10 ng/mL EGF (Sigma Chemical, St. Louis, MO, USA) for one day. Subsequently, cells were incubated with 20 ng/mL of EGF and 10 µM of all-trans retinoic acid (ATRA) for 1 week. ATRA was then removed and the cells were exposed to GRM supplemented with 20 ng/mL of EGF for 25 days. At day 28, the spheres were plated in Petri dishes coated with 1:30 Matrigel for 1 week and cultured on GRM supplemented with 20 ng/mL EGF. For terminal differentiation, at day 35, OPCs were seeded on poly-l-lysine and human laminin (Sigma Chemical) coated plates.

### 4.6. Sub-Cellular Protein Fractionation

Nuclear protein extraction from cells that undergo the differentiation protocol to oligodendrocytes was performed by using Nuclear Extraction Kit (Panomics, San Diego, CA, USA) according to the manufacturer’s instructions.

### 4.7. Isolation and Culture of Rat Spinal Cord Astrocytes

A culture enriched population of astrocytes from intact adult rat spinal cord was obtained as previously described [43]. Briefly, after spinal cord extraction and removal of meninges the tissue was incubated with 4 mg/mL papain in DMEM for 2 h at 37 °C. After mechanical homogenization, the sample was filtered by a 40 µm filter. After centrifugation for 5 min at 1500 rpm, cells were resuspended in 6 mL of DMEM with 10% FBS and transferred to the top of OptiPrep gradient (Sigma). The preparation was centrifuged for 15 min at 2200 rpm and cells from the appropriate portion were collected, centrifuged again at 2000 rpm 5 min and plated on previously coated poly-l-lysine plates. Samples were prepared for further immunocytochemistry and analysis by confocal microscopy (Leica, Wetzlar, Germany).

### 4.8. Real-Time Polymerase Chain Reaction (Taqman)

Total RNA obtained from adult female Sprague Dawley rats by using NucleoSpin RNA/prot (Macherey Nagel, Düren, Germany) was reverse-transcribed using TaqMan reverse transcription reagents (Applied Biosystems, Foster City, CA, USA) following manufacturer’s instructions. As template, 40 ng of cDNA from target and housekeeping genes was prepared independently for each TaqMan reaction. Each reaction was performed in duplicate in three independent experiments. The MGB assay on demand TaqMan probes (Applied Biosystems) for Cx50 (Rn00596378_s1) were used and results were referred to *GAPDH* (Rn01775763_g1) (housekeeping gene). The comparative threshold cycle (CT) method was used to calculate the relative expression [44].

### 4.9. Western Blotting Analysis

Cells were collected and proteins extracted by using Lysis Buffer (50 mM Tris-HCl, pH 7.5, 150 mM NaCl, 0.02% NaN_3_, 0.1 SDS, 1% NP40, 1 mM EDTA, 2 µg/mL leupeptine, 2 µg/mL aprotinine, 1 mM PMSF, 1 × Protease Inhibitor Cocktail (Roche Diagnostics, San Diego, CA, USA)). Equal amounts of protein extracts (20 µg) were loaded onto a 10% SDS-polyacrylamide gel and resolved by standard SDS-PAGE. Proteins were electrophoretically transferred onto PVDF membranes. Membranes were blocked with 5% skimmed milk in PBST for 60 min and tested overnight with specific antibodies at dilution 1:500 against, Cx50 (Invitrogen), Olig1 (Chemicon International, Billerica, MA, USA), GFAP (Dako, Glostrup, Denmark), Tuj1 (Covance, Princeton, NJ, USA) and β-actin at dilution 1:5000 (Sigma Chemical, USA), which was used as loading control. Subsequently, membranes were incubated with rabbit anti-mouse or rabbit anti-goat horseradish peroxidase-conjugated secondary antibody (1:5000) (Sigma Chemical). Blots were visualized by the ECL detection system (Amersham, Buckinghamshire, UK).

### 4.10. Immunocytochemistry

Spinal cord tissue was isolated from all animals after intracardial perfusion with 4% PFA solution and then cryopreserved and sectioned at 10 μm. Cells and tissues were fixed with 4% PFA at room temperature for 15 min, permeabilized with 0.1% Triton X-100, and subsequently blocked with 10% of FBS. Incubation with or without (as controls for secondary antibodies non-specific binding) primary antibodies was performed overnight (1:200) at 4 °C: Cx50 (Invitrogen), GFAP (Dako), Olig1 (Chemicon International), Tuj1 (Covance) or RIP (Chemicon International). After washing secondary antibodies, Oregon green 488 goat anti-mouse IgG, or Alexa Fluor 647 mouse anti-rabbit 1:400 (Thermo Fisher Scientific, Waltham, MA, USA) were incubated for 1 h at room temperature. Signals were visualized by Confocal microscopy (Leica).

### 4.11. Statistical Analysis

Statistical comparisons were assessed by the Student’s *t*-test. All *p* values were derived from a two-tailed statistical test using the SPSS 11.5 Software. A *p*-value < 0.05 was considered statistically significant.

## 5. Conclusions

Cx50, in spontaneous differentiation conditions, favors the glial cell fate and impairs neural cell differentiation in neural precursor cells (epSPC) from adult spinal cord. epSPC activated by SCI (epSPCi) exhibit low levels of Cx50 and GFAP expression. epSPCi transplantation creates a more permissive environment for axonal survival and re-growth. Therefore, modulation of Cx50 expression after SCI might enable a greater control over the neurogenic capacity of activated epSPCi for functional neural regeneration.
